# Differences in Adipose Tissue and Lean Mass Distribution in Patients with Collagen VI Related Myopathies Are Associated with Disease Severity and Physical Ability

**DOI:** 10.3389/fnagi.2017.00268

**Published:** 2017-08-08

**Authors:** M. A. Rodríguez, Luís M. Del Rio Barquero, Carlos I. Ortez, Cristina Jou, Meritxell Vigo, Julita Medina, Anna Febrer, Marta Ramon-Krauel, Jorge Diaz-Manera, Montse Olive, Laura González-Mera, Andres Nascimento, Cecilia Jimenez-Mallebrera

**Affiliations:** ^1^Neuromuscular Unit, Department of Neuropaediatrics, Institut de Recerca Sant Joan de Déu, Hospital Sant Joan de Déu Barcelona, Spain; ^2^Department of Medical Imaging, Hospital Sant Joan de Déu Barcelona, Spain; ^3^CETIR Centre Mèdic Barcelona, Spain; ^4^Department of Pathology, Hospital Sant Joan de Deu Barcelona, Spain; ^5^Center for Biomedical Research on Rare Diseases, Instituto de Salud Carlos III Madrid, Spain; ^6^Department of Rehabilitation and Physical Medicine, Hospital Sant Joan de Deu Barcelona, Spain; ^7^Department of Endocrinology, Institut de Recerca Sant Joan de Deu, Hospital Sant Joan de Deu Barcelona, Spain; ^8^Neuromuscular Disorders Unit, Department of Neurology, Hospital de la Santa Creu i Sant Pau, Universitat Autónoma de Barcelona Barcelona, Spain; ^9^Department of Pathology and Neuromuscular Unit, Bellvitge Biomedical Research Institute-Hospital de Bellvitge, Hospitalet de Llobregat Barcelona, Spain; ^10^Department of Neurology, Hospital de Viladecans Barcelona, Spain

**Keywords:** myopathy, collagen VI, body composition, sarcopenia, obesity, adiponectin, leptin, physical ability

## Abstract

Mutations in human collagen VI genes cause a spectrum of musculoskeletal conditions in children and adults collectively termed collagen VI-related myopathies (COL6-RM) characterized by a varying degree of muscle weakness and joint contractures and which include Ullrich Congenital Muscular Dystrophy (UCMD) and Bethlem Myopathy (BM). Given that collagen VI is one of the most abundant extracellular matrix proteins in adipose tissue and its emerging role in energy metabolism we hypothesized that collagen VI deficiency might be associated with alterations in adipose tissue distribution and adipokines serum profile. We analyzed body composition by means of dual-energy X-ray absorptiometry in 30 pediatric and adult COL6-RM myopathy patients representing a range of severities (UCMD, intermediate-COL6-RM, and BM). We found a distinctive pattern of regional adipose tissue accumulation which was more evident in children at the most severe end of the spectrum. In particular, the accumulation of fat in the android region was a distinguishing feature of UCMD patients. In parallel, there was a decrease in lean mass compatible with a state of sarcopenia, particularly in ambulant children with an intermediate phenotype. All children and adult patients that were sarcopenic were also obese. These changes were significantly more pronounced in children with collagen VI deficiency than in children with Duchenne Muscular Dystrophy of the same ambulatory status. High molecular weight adiponectin and leptin were significantly increased in sera from children in the intermediate and BM group. Correlation analysis showed that the parameters of fat mass were negatively associated with motor function according to several validated outcome measures. In contrast, lean mass parameters correlated positively with physical performance and quality of life. Leptin and adiponectin circulating levels correlated positively with fat mass parameters and negatively with lean mass and thus may be relevant to the disease pathogenesis and as circulating markers. Taken together our results indicate that COL6-RM are characterized by specific changes in total fat mass and distribution which associate with disease severity, motor function, and quality of life and which are clinically meaningful and thus should be taken into consideration in the management of these patients.

## Introduction

COL6-related myopathies (COL6-RM) are caused by mutations in COL6A genes (*COL6A1, COL6A2*, and *COL6A3*) and biochemical defects in collagen VI. They represent a continuum of clinical phenotypes from early severe forms (Ullrich Congenital Muscular Dystrophy, UCMD) to milder presentations (Bethlem Myopathy, BM) and intermediate phenotypes (Cruz et al., 2016). Collagen VI is an ubiquitously expressed extracellular matrix protein which is particularly abundant in adipose tissue where it increases with adipogenesis and localizes surrounding the adipocyte (Nakajima et al., 2002; Khan et al., 2009; Pasarica et al., 2009). Furthermore, the expression of collagen VI genes is regulated positively by glucose levels and negatively by PPAR-γ agonists and leptin (Muona et al., 1993; Khan et al., 2009; Dankel et al., 2014; McCulloch et al., 2015). There is increasing evidence that collagen VI is an important regulator of adipose tissue function and several recent studies have described the implication of collagen VI in the metabolic context. For example, COL6A3 mRNA levels are associated with insulin-resistance (Spencer et al., 2010; Dankel et al., 2014) and obesity in mice and humans (Khan et al., 2009; McCulloch et al., 2014). In line with this, lack of collagen VI has been related to a reduction of fat accumulation and improvement in insulin sensitivity and glucose clearance in obese mice (Khan et al., 2009).

Our hypothesis was that collagen VI deficiency, in addition to a loss of muscle mass, is characterized with changes in the total amount and distribution of adipose tissue. These changes are likely to contribute to the deterioration of motor abilities and quality of life and may predispose patients to metabolic complications and other health issues. Increased fat mass and alterations in the distribution of adipose tissue as well as low muscle mass represent a health risk factor for metabolic and cardiovascular diseases such as diabetes (Franks et al., 2010; Burrows et al., 2017).

Despite being considered only a storage tissue, adipose tissue is an endocrine organ able to synthesize and release signaling molecules called adipokines such adiponectin, leptin, and retinol binding protein 4 (RBP4) (Antuna-Puente et al., 2008).

Adiponectin is an adipokine highly present in blood relative to other adipose tissue hormones. It is directly correlated with insulin sensibility through its action regulating glucose levels and lipid oxidation (Shehzad et al., 2012; Caselli, 2014).

On the other hand, leptin levels are directly correlated with adiposity (Oral and Chan, 2010) and play an important role in satiety, affecting food intake and metabolism (Kotnik et al., 2015). Recent studies report that leptin levels regulate the expression of collagen VI α3 chain (COL6A3) (McCulloch et al., 2014).

Similarly, retinol binding protein-4 (RBP4) is an adipocyte-derived hormone which contributes to pathogenesis of type 2 diabetes and had been shown to be elevated in insulin-resistance both in mouse models and humans (Yang et al., 2005).

Previous gene expression studies from our laboratory (Paco et al., 2013) showed that several adipokines were over-expressed in UCMD muscle relative to control muscle, in particular, the genes encoding for adiponectin (9.6-fold change), leptin (5.1-fold change), and RBP4 (19.6-fold change) which were amongst the most over-expressed genes. However, the circulating levels of these hormones in patients with collagen VI related myopathies has not been investigated.

The aim of the present study was to analyze changes in total amount and regional distribution of fat and lean tissue using dual-energy X-ray absorptiometry (DXA) in a population of pediatric and adult COL6-RM myopathy patients representing a range of severities and to correlate the findings with various clinical parameters and with circulating levels of adipokines.

## Materials and Methods

### Ethics Statement

This study was carried out in accordance with the recommendations of the Hospital Sant Joan de Déu Ethics Committee. Written informed consent was obtained from patients and/or their parents or guardians in accordance with the Declaration of Helsinki. The protocol was approved by the Hospital Sant Joan de Déu Ethics Committee.

Serum samples were stored in the Biobank of the Hospital Sant Joan de Déu.

### Patients

A total of 30 patients with COL6-RM were included in the study. They were seen at the Hospital Sant Joan de Déu, Hospital de la Santa Creu i Sant Pau, and Hospital de Bellvitge in Barcelona. Patients were diagnosed by clinical exam and medical history and analysis of collagen VI in muscle biopsy and/or dermal fibroblasts. In all patients diagnosis was confirmed at the genetic level.

Patients were grouped according to clinical criteria as previously described (Nadeau et al., 2009; Foley et al., 2013; Deconinck et al., 2014; Meilleur et al., 2014; Cruz et al., 2016).

Early onset/severe (UCMD): Those patients that never achieved ambulation or lost ambulation before the age of 10 (*n* = 6). Mean age was 8.6 years at the time of study (age range 2–17 years), 5 males and 1 female.

Intermediate COL6-RM: children who have achieved ambulation and are currently walking beyond the age of 10 but cannot run or hop, and/or have developed respiratory insufficiency and are likely to loose ambulation before the age of 19 (*n* = 8). Mean age was 10.6 (age range 5–16 years), 5 males and 3 females. Here, we included three patients aged 5, 6, and 7 at the time of the study who are ambulant [6-min-walking test (6MWT) above 300 m] but cannot run or jump and are likely to remain ambulant beyond 10 years of age.

Bethlem Myopathy: Individuals who achieve and maintain ambulation into adulthood (*n* = 16). Mean age: 33.1 years (age range: 11–59 years), 6 males and 10 females. Within this group we included four teenagers between 11 and 17 years old at the time of the study who are mildly affected (able to run and have a 6MWT above 400 m and have not developed respiratory insufficiency).

Body composition parameters in UCMD and intermediate COL6-RM patients were compared with patients with genetically confirmed Duchenne Muscular Dystrophy (*n* = 31, age range 5–18) which were either ambulant (*n* = 17) or non-ambulant (14) at the time of DXA analysis.

### Body Composition by DXA

Body composition measurements were made by DXA with a General Electric Healthcare unit, Prodigy model, software version 12.3. Distinctions made in each body region provided an analysis of the composition in a model of three components: skeletal mineral content (BMC), fat body mass (FM), and lean body mass (LM).

For scanning, patients were positioned supine, following the manufacturer’s recommendations for this type of exploration. However, the technique had to be adapted to the particular characteristics of our patients. In all cases total measurements were estimated on the values of the right half-ibody. Regions explored in patients were: whole body, upper and lower extremities, trunk, lower abdominal region of the trunk (limited by iliac crests and the upper limit of the ribs) and the sector of the hips, the latter two regions designated android and gynecoid, respectively. With results of whole-body and regional measurements the following ratios were calculated: fat mass index (FMI) [FM/height (Nakajima et al., 2002)] and lean mass index (LMI) [LM/height (Nakajima et al., 2002)]. Appendicular fat free mass index (AFFMI) was calculated as the sum of lean mass in lower extremities and upper extremities/height (Nakajima et al., 2002). Relationships between the fatty tissue and lean tissue of the lower extremities and upper extremities, adjusted for the size of the subject, were calculated.

Appendicular fat free mass index was used to define a state of sarcopenia when it was below 2 standard deviations from the mean value observed in the reference population for the same age and sex (Baumgartner et al., 1998). Sarcopenic obesity was defined according to Baumgartner et al. (2004) as a decrease in AFFM of two or more 2 standard deviations accompanied by increase in fat mass above 27% in male and 40% in female patients, respectively.

### Enzyme-Linked Immunosorbent Assay

Enzyme-linked immunosorbent assay (ELISA) was used for detection of adiponectin high molecular weight (HMW adiponectin), leptin and binding retinol protein 4 (RBP4) levels using commercial ELISA kits #EZHMWA-64K, #EZHL-80SK (Millipore, Temecula, CA, United States) and DRB400 (R&D systems, Minneapolis, MN, United States) respectively, in serum samples of patients which were collected the same day of DXA analysis. Plates were incubated and treated as described in the manufacturer’s protocol and then the absorbance was detected at 450 nm by EMax^®^ Microplate Reader (Molecular Devices, Sunnyvale, CA, United States). Values were compared with the standard curve and concentration of each sample was determined (ng/mL).

### Patient Assessment of Physical Performance and Quality of Life

As outcome measures of physical performance in ambulant patients we applied the Motor Function Measure 32 (MFM32), the North Star Ambulatory Assessment (NSAA), the upper limb scale (PULL.85), 6MWT, timed function tests (time taken to rise from floor, run/walk 10 m, climb four standard-sized stairs, and descend four standard-sized stairs) (McDonald et al., 2013). For non-ambulant patients we applied the Egen Klassifikation 2 (EK2) and the upper limb scales (PULL.85) since these have been validated and shown to be feasible and reliable in this group of patients (Meilleur et al., 2014). The neuromuscular module of the pediatric quality of life (PedsQL) scale was used to obtain an indication of patient (and parent) perceived quality of life.

### Statistical Analysis

To assess changes in body composition in patients, measurements of each disease group were compared with those of healthy Spanish subjects of the same sex and age which were taken as reference values for each of the variables. These data from healthy controls (consisting of a total of 1300 individuals between 7 and 80 years old grouped by sex and age) were collected by Dr. Luis del Río. Body mass index (BMI) of patients under 20 years of age was compared with WHO database using the “WHO AnthroPlus Software Manual for Personal Computers: Software for assessing growth of the world’s children and adolescents. Geneva: WHO, 2009^1^.”

Statistically significant changes for each variable in the disease population were studied by Z-score which was calculated with the formula *z* = (*x* - μ)/σ; where *x* represents the value in the patient; μ is the mean value in the reference population, and σ is the value of the standard deviation in the reference population. A change in Z-score value largest than 2 standard deviations was considered statistically significant. The positive or negative sign of Z indicated the direction of the deviation.

Pearson correlation coefficient was calculated using SPSS software (SPSS 19.0 (Armonk, NY, United States: IBM Corp) to investigate associations between each body composition measurement and the different functional scores, timed-tests, and adipokines levels.

## Results

### Body Composition

**FIGURE 1:**
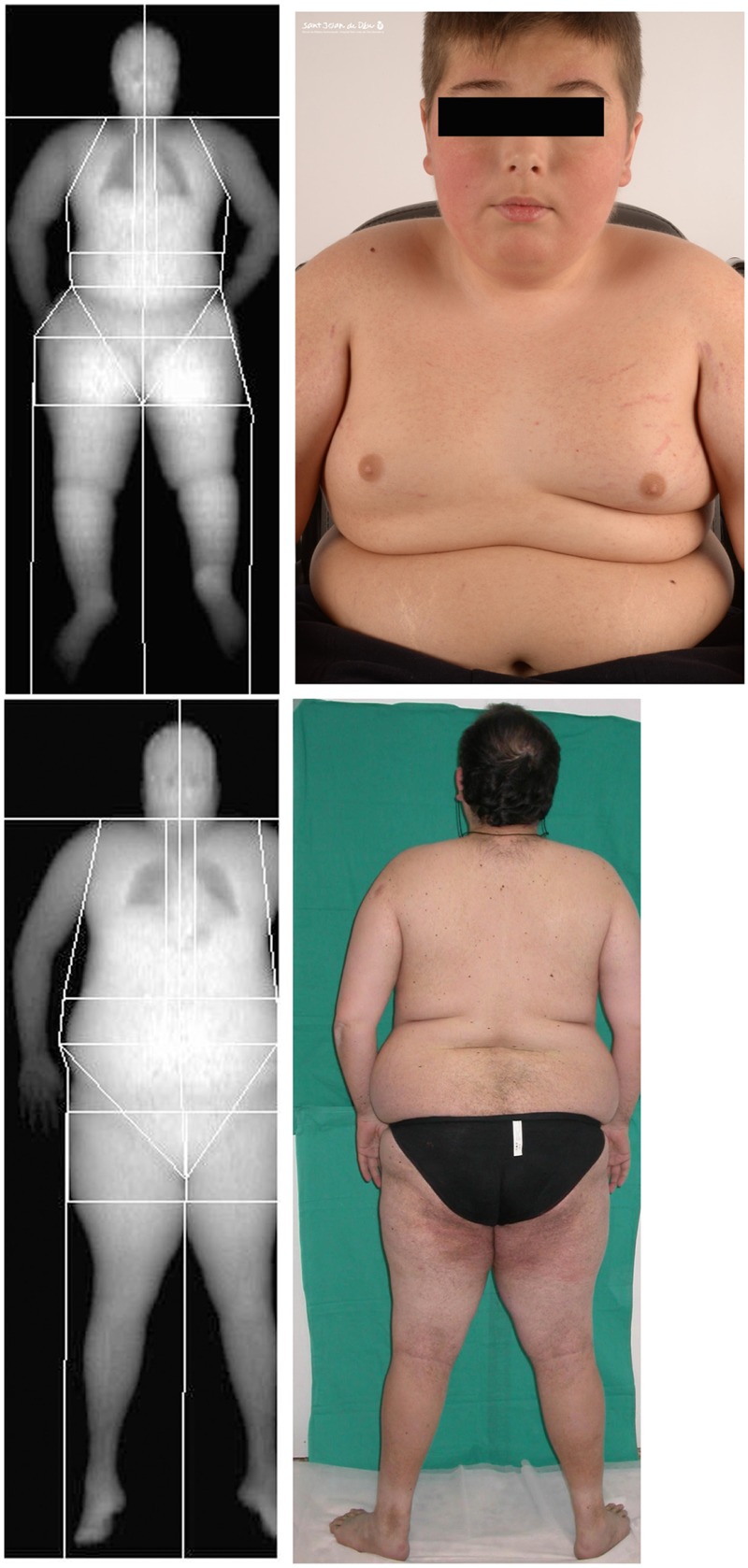
Representative dual-energy X-ray absorptiometry (DXA) scan showing the soft tissues in the different regions analyzed for one of the children with Ullrich Congenital Muscular Dystrophy (UCMD), Top, and an adult patient with Bethlem Myopathy (BM), Bottom, and their corresponding clinical images. Written informed consent was obtained for the publication of this image.

Body composition measurements were made by DXA. Regions explored included whole body, upper and lower extremities, trunk, lower abdominal region of the trunk (android), and the sector of the hips (gynecoid) (**Figure 1**).

**FIGURE 2:**
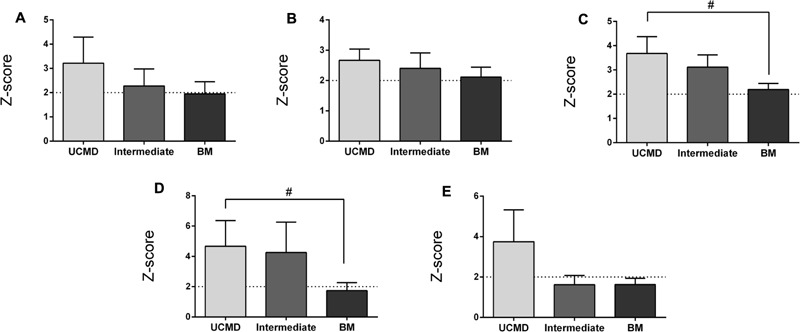
Changes in amount of fat mass by DXA in UCMD, intermediate phenotype and BM patients in whole body normalized to height squared (fat mass index, FMI) **(A)**, gynecoid region **(B)**, legs **(C)**, trunk **(D)**, and android region **(E)**. Results are expressed as mean on Z-score for each group. Threshold of significance was considered when Z-score ≥ 2 or Z-score ≤ –2 and was indicated with dotted line. ^#^*P* ≤ 0.05.

The FMI (see “Materials and Methods”) was on average significantly elevated in UCMD and intermediate groups but normal in BM patients (**Figure 2A**). In contrast the mean BMI was within normal limits in all three groups relative to the Spanish reference population (data not shown). The percentage of fat in the legs and the amount of fat accumulated in the gynecoid region were also increased in all three groups being highest in the more severe patients (UCMD) followed by intermediate and then BM patients (**Figures 2B,C**). The amount of fat in the trunk was elevated in UCMD and intermediate patients (**Figure 2D**) whereas the amount of fat in the android region was elevated in UCMD patients only (**Figure 2E**). Within the intermediate group, Z-scores were significantly higher in female patients than in their male counterparts. We could not analyze sex differences in UCMD patients since there was only one female patient. There were no significant sex differences in BM patients.

**FIGURE 3:**
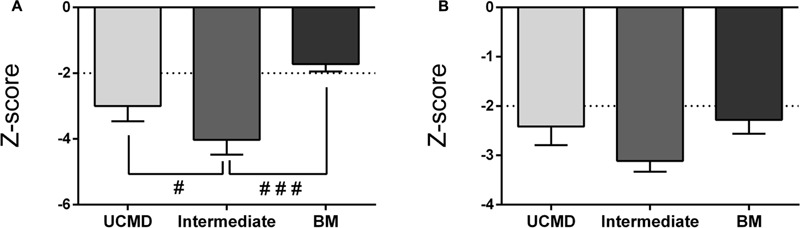
Changes in amount of lean mass by DXA in UCMD, intermediate phenotype and BM patients in whole body normalized to height squared (lean mass index, LMI) **(A)** and appendicular free fat mass normalized to height squared (AFFMI) **(B)**. Results are expressed as mean on Z-score for each group. Threshold of significance was considered when Z-score ≥ 2 or Z-score ≤ –2 and was indicated with dotted line. ^#^*P* ≤ 0.05 and ^###^*P* ≤ 0.001.

The LMI was significantly reduced in UCMD and intermediate groups but not in the BM group whereas the AFFMI was significantly reduced in all three groups. For both parameters the lowest Z-score was found in intermediate patients (**Figures 3A,B**). There were no significant sex differences regarding these lean mass parameters (data not shown).

Appendicular fat free mass index is used as an indicator of sarcopenia which is a condition characterized by loss of muscle mass and either low muscular strength or low physical performance (Cruz-Jentoft et al., 2010).

Sarcopenia has been previously used to describe the extent of decline of muscle mass in patients with various forms of muscular dystrophy and in adults with COL6-RM (Miscione et al., 2013; Merlini et al., 2014). According to established criteria (AFFMI below a Z-score of 2), (Baumgartner et al., 2004) 66% of UCMD patients, 100% of intermediate COL6-RM patients, and 62% of BM patients were sarcopenic.

**FIGURE 4:**
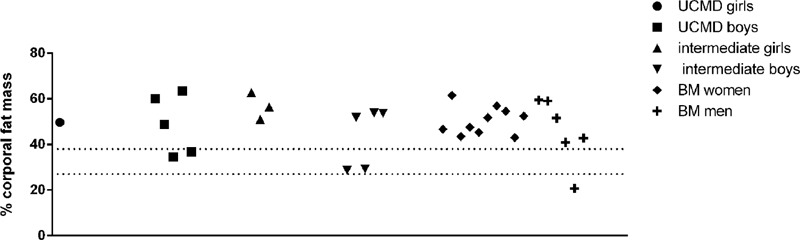
Representation of the percentage of body fat mass in UCMD, intermediate phenotype and BM patients divided by sex. The percentage of body fat mass of each patient was calculated as the ratio between the value of the total amount of body fat mass and the weight of the patient. The value obtained was multiplied by 100. Dotted lines indicate obesity threshold based on Baumgartner (2000) (>27% for men and >40% in women).

All patients (children and adult) except one BM patient (who was not sarcopenic) had an overall percentage of fat mass above the obesity threshold (28% for women and 40% for men) (**Figure 4**). Therefore, all sarcopenic patients were also obese as previously described in adults with COL6-RM (Miscione et al., 2013).

To investigate the effect of ambulation in the observed changes we compared the body composition of children in the UCMD (all non-ambulant) and intermediate (all ambulant) groups with those of ambulant (*n* = 17) and non-ambulant DMD (*n* = 14) children, respectively, within the same age range. Although both forms of muscular dystrophy are different in many respects (age of onset, pattern of weakness, etc.) both lead to early loss of ambulation and a decline in muscle mass and function (Merlini et al., 2014).

**FIGURE 5:**
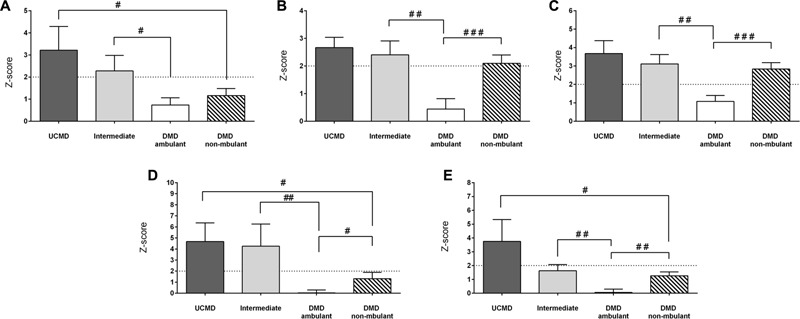
Changes in amount of fat mass by DXA in UCMD, intermediate phenotype and DMD ambulant and non-ambulant patients in whole body normalized to height squared (FMI) **(A)**, gynecoid region **(B)**, legs **(C)**, trunk **(D)**, and android region **(E)**. Results are expressed as mean on Z-score for each group. Threshold of significance was considered when Z-score ≥ 2 or Z-score ≤ –2 and was indicated with dotted line. ^#^*P* ≤ 0.05, ^##^*P* ≤ 0.01, and ^###^*P* ≤ 0.001.

We observed that FMI, fat mass in the trunk and android regions were normal in both ambulant and non-ambulant DMD patients whereas the percentage of fat in the legs and the amount of fat in the gynecoid regions were significantly elevated in non-ambulant DMD (Z-score > 2) (**Figure 5**) relative to the reference population.

The FMI, fat in the trunk and android fat were significantly higher in UCMD (non-ambulant patients) than in non-ambulant DMD patients. All fat parameters were significantly higher in intermediate COL6-RM patients than in ambulant DMD children (**Figure 5**).

**FIGURE 6:**
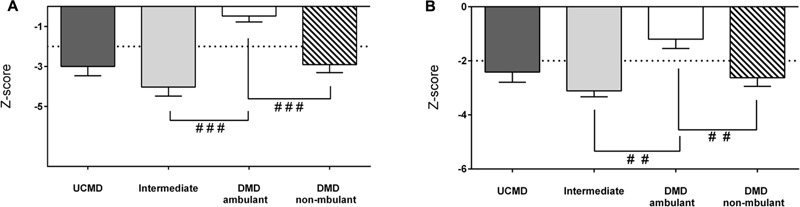
Changes in amount of lean mass by DXA in UCMD, intermediate phenotype and DMD ambulant and non-ambulant patients in whole body normalized to height squared LMI **(A)** and appendicular free fat mass normalized to height squared (AFFMI) **(B)**. Results are expressed as mean on Z-score for each group. Threshold of significance was considered when Z-score ≥ 2 or Z-score ≤ –2 and was indicated with dotted line. ^#^*P* ≤ 0.05, ^##^*P* ≤ 0.01, and ^###^*P* ≤ 0.001.

With regards to lean mass, the LMI and AFFMI were normal in ambulant DMD children and reduced in non-ambulant DMD children relative to the reference population (**Figure 6**).

The LMI and the AFMMI were significantly lower in intermediate COL-6 RM patients than in ambulant DMD patients (and in non-ambulant DMD vs. ambulant DMD children) (**Figure 6**). However, there were no significant differences between UCMD and non-ambulant DMD groups.

### Adipokines Profile

**FIGURE 7:**
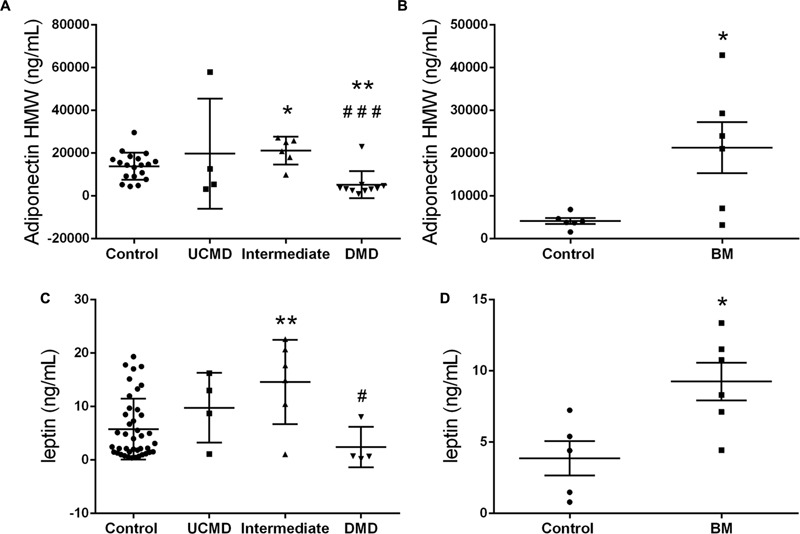
High molecular weight Adiponectin serum levels in UCMD and intermediated phenotype patients relative to controls and DMD patients **(A)** and in BM patients relative to adult controls **(B)**. Leptin serum levels in UCMD and intermediated phenotype patients relative to controls and DMD patients **(C)** and in BM patients relative to adult controls **(D)**. Serum samples were analyzed by ELISA technique. Adipokine levels are expressed as mean ± SEM for each group of patients. ^∗^*P* ≤ 0.05 and ^∗∗^*P* ≤ 0.01 relative to controls and ^#^*P* ≤ 0.05 and ^###^*P* ≤ 0.001 relative to intermediated phenotype.

Serum leptin, HMW adiponectin and RBP4 were analyzed by ELISA in sera from UCMD (*n* = 4), intermediate COL6-RM (*n* = 6), and BM patients (*n* = 6) and compared with either pediatric controls (*n* = 19) or adult controls (*n* = 6) and with DMD children as a disease control group (*n* = 10). We found a significant increase of serum adiponectin and leptin levels in patients within the intermediate and milder BM phenotype but not in the more severe UCMD patients. In contrast, adiponectin HMW was significantly reduced in DMD children (**Figure 7**). We did not observe any significant change in RBP4 in any of the groups analyzed.

### Correlation Analysis with Physical Ability and Quality of Life

We then looked for possible associations between the various parameters analyzed. A correlation was considered large if *r* > 0.65, medium if *r* between 0.5 and 0.65, and small when *r* is below 0.5.

In non-ambulant UCMD patients the largest and significant correlations were found between the fat mass parameters and the EK2 score (the lower the better physical performance) as well as with the PUL score (the higher the better performance) (**Table 1**). Surprisingly, in both cases the higher the value of the fat indices the better the performance. The LMI showed a strong correlation with the PUL score. There was also a positive correlation between fat mass indices and the score for the quality of life questionnaire (the higher the worse self-perception of quality of life).

**Table 1:** Correlations in Ullrich Congenital Muscular Dystrophy (UCMD) patients expressed as values of Pearson’s correlation coefficient (*r*) between variables of body composition expressed in Z-score (lines) and variables of motor function, quality of life or adipokine levels (columns).

	EK2	PULL	Peds QL	Peds QL Parents	Leptin	HMW adiponectin
FMI	-0.83*	0.77	0.72	0.73	0.57	0.73
Fat mass trunk	-0.88*	0.78	0.67	0.54	0.5	0.66
Android	-0.69	0.54	0.44	0.67	0.29	0.95
% Fat mass legs	-0.86*	0.4	0.99	0.64	0.46	0.87
Gynecoid	-0.73	-0.45	0.33	0.01	0.63	0.76
LMI	0.55	0.86	0.57	-0.12	-0.62	-0.66
AFFMI	0.32	0.35	0.52	0.25	-0.04	-1.00**

*Statistical significance was tested with two-tailed *t* student ^∗^*P* ≤ 0.05 and ^∗∗^*P* ≤ 0.001.*

In the intermediate group (**Table 2**) strong and significant correlations were found between the percentage of fat in legs, android, and gynecoid regions and the time to run 10 m. The amount of fat in the trunk correlated negatively and significantly with the PUL score. There was a large and significant correlation between the LMI and the NSAA and MFM32 scores. In contrast, the AFFMI did not show any large correlation with any of the body composition parameters in this group of patients.

**Table 2:** Correlations in intermediate phenotype patients expressed as values of Pearson’s correlation coefficient (*r*) between variables of body composition expressed in Z-score (lines) and variables of motor function, quality of life or adipokine levels (columns).

	6MWT	Raise from floor	Climb up 4 stairs	Climb down 4 stairs	10 m	MFM	NSAA	PUL	PedsQL	PedsQL parents	Leptin	HMW adiponectin
FMI	-0.36	-0.17	0.68	0.64	0.68	-0.26	-0.26	0.01	0.31	0.09	0.56	0.49
Fat mass trunk	-0.11	0.83	0.14	0.12	0.26	-0.36	-0.39	-0.96*	0.78*	0.75*	0.62	0.48
Android	-0.41	0.36	0.67	0.63	0.78*	-0.47	-0.54	-0.29	0.48	0.33	0.8	0.29
% Fat Mass Legs	-0.52	-0.1	0.7	0.67	0.82*	-0.35	-0.4	-0.13	0.42	0.25	0.64	0.31
Gynecoid	-0.56	0.33	0.78*	0.75	0.86*	-0.56	-0.62	-0.29	0.53	0.36	0.75	0.25
LMI	0.55	-0.59	-0.49	-0.5	-0.54	0.91*	0.84*	0.48	-0.46	-0.53	-0.2	0.64
AFFMI	0.29	-0.21	0.05	-0.01	0.1	0.32	0.26	0.02	-0.04	-0.11	0.54	0.4

*Statistical significance was tested with two-tailed *t* student ^∗^*P* ≤ 0.05.*

The quality of life questionnaire score showed a positive and significant correlation with the Z-score of the fat in the trunk (in children and parents).

In the BM group the percentage of fat mass in legs correlated negatively with the 6MWT and the MFM and NSAA scores and positively with the time to walk/run 10 m and the time to raise from the floor, although none of these reached statistical significance (**Table 3**). There was a strong and significant negative correlation between the amount of fat in the android region and the MFM score.

**Table 3:** Correlations in BM patients expressed as values of Pearson’s correlation coefficient (*r*) between variables of body composition expressed in Z-score (lines) and variables of motor function, quality of life or adipokine levels (columns).

	6MWT	Raise from floor	Climb up 4 stairs	Climb down 4 stairs	10 m	MFM	NSAA	PUL	PedsQL	PedsQL parents	Leptin	HMW adiponectin
FMI	-0.30	0.78	0.45	0.32	0.48	-0.53	-0.19	-0.15	-0.17	0.98	0.51	0.37
Fat mass trunk	-0.01	0.73	0.10	0.09	0.29	-0.60	-0.46	0.28	-0.51	0.69	0.24	-0.03
Android	-0.55	0.77	0.48	0.51	0.65	-0.82*	-0.69	-0.12	-0.06	0.99	0.63	0.23
% Fat mass legs	-0.67	0.71	0.53	0.55	0.67	-0.77	-0.74	-0.25	0.02	0.78	0.56	0.35
Gynecoid	-0.38	0.71	0.48	0.38	0.52	-0.55	-0.21	-0.28	-0.06	0.72	0.65	0.41
LMI	0.40	-0.24	-0.64	-0.48	-0.54	0.02	-0.33	0.65	-0.17	0.01	-0.63	-0.24
AFFMI	0.15	-0.25	-0.56	-0.34	-0.41	-0.12	-0.53	0.56	-0.12	-0.17	-0.77	-0.14

*Statistical significance was tested with two-tailed *t* student ^∗^*P* ≤ 0.05.*

There were several large and significant correlations between the body composition parameters and the circulating levels of leptin and adiponectin. In the UCMD group, levels of HMW adiponectin correlated positively with the indices of fat mass and negatively with the amount of lean mass (in particular with the AFFMI). A similar tendency was observed in the other groups (**Tables 1–3**).

## Discussion

Herein we report a detailed description of changes in body composition by means of DXA in children with COL6-RM and how they relate to physical performance.

We found a significant increase in total body fat mass and/or in the amount of fat in specific body regions which depended on severity of the phenotype.

All fat mass parameters were higher in the most severe UCMD patients than in patients with the intermediate phenotype and patients with BM. The amount of fat in the android region was increased in UCMD patients whereas it was comparable to the reference population in patients with the intermediate phenotype (although differences between both groups were not significant). Furthermore there was a statistically significant difference in the amount of fat in the trunk and legs between UCMD and BM patients.

Our results suggest that in COL6-RM children, the increased adiposity cannot be ascribed solely to the ambulatory status. When we analyzed body composition in DMD patients the results were clearly different. In non-ambulant DMD patients four out of the five fat mass parameters analyzed were normal or only marginally increased and in ambulant DMD patients none of the fat mass parameters were elevated in comparison with the reference population and in contrast to children with collagen VI deficiency.

Furthermore, all indices of adiposity were higher in UCMD patients than in non-ambulant DMD patients and in intermediate COL6-RM patients than in ambulant DMD children. The difference between UCMD patients and non-ambulant DMD children was statistically significant for the central regions (trunk and android) suggesting that these body areas are more prone to fat accumulation in association with collagen VI defects.

The deviation from normal of the total lean mass was larger in UCMD and intermediate phenotype patients than the deviation of the lean mass in the extremities probably reflecting the predominant involvement of axial and abdominal muscles. In contrast the LMI was normal in BM patients whereas the AFFMI was mildly reduced. In both cases the Z-scores were lower in patients within the intermediate category (ambulant) than in patients with UCMD (non-ambulant). This suggests that pathways triggered by physical activity may determine the extent of muscle loss. On the other hand, the loss of muscle mass in the most severe UCMD patients (even in the youngest patients who never walked) indicates that it is a feature inherent to the disease in line with the observed muscle fiber atrophy that we and others have described in muscle biopsies of very young patients (Schessl et al., 2008; Paco et al., 2012).

The LMI and AFFMI were normal in ambulant DMD children and reduced in non-ambulant DMD children relative to the reference population. This is in agreement with previous findings of significantly lower mean mass in non-ambulant adult muscular dystrophy patients than in ambulant ones (Merlini et al., 2014). Furthermore, the LMI and the AFMMI were significantly lower in intermediate patients than in ambulant DMD patients (and in non-ambulant DMD versus ambulant DMD children).

These observations suggest that in dystrophin deficiency the loss of muscle mass is associated with loss of ambulation whereas in UCMD this is not the case.

In all three groups of patients sarcopenia co-existed with obesity as previously described in adult patients (Merlini et al., 2014). Also, similar to what other authors have reported, there was no deviation from normal in the mean BMI which may be explained by the loss of muscle mass and the fatty infiltration of skeletal muscle (Toni et al., 2014) and which highlights the usefulness of the determination of total fat mass by DXA.

There was a clear association between various indices of fat mass and physical performance. In particular, within the group of patients with the intermediate phenotype the correlation analysis showed several informative associations. This may be partly due to the wider breadth of tests of physical performance that can be applied in this group of patients compared to the non-ambulant UCMD group. In particular, the amount of fat accumulated in the android, gynecoid regions, and legs were very good indicators of worse physical performance in particular when using the 10 m test. In contrast, the overall lean mass (LMI) correlated strongly and significantly with the NSAA and MFM scores. The fat mass in the central body region (trunk) showed a very strong and significant association with the quality of life as perceived by the patients and their parents (the higher the amount of fat the worse quality of life score).

Thus, these observations suggest that body composition parameters as measured by DXA can be used as quantitative and objective markers of physical performance although further validation in larger cohorts is required. These results can also help toward selecting the most appropriate outcome measures in each subgroup of patients according to their severity for a more refined analysis of COL6-RM.

There are two other studies looking at body composition by DXA in patients with collagen VI deficiency although both look at the same cohort composed of one adult UCMD and seven adult BM patients (Miscione et al., 2013; Toni et al., 2014). In those studies they use as the reference population a cohort of healthy individuals from the United States (Kelly et al., 2009). In our study, we have used as a reference a Spanish healthy population removing thus variability due to differences in diet and lifestyle between different populations/countries. Finally, in our study we provide additional data on regional fat accumulation.

Our previous microarray data demonstrated an increase in the mRNA levels of the main adipokines secreted by adipose cells (leptin, adiponectin, and RBP4) in skeletal muscle biopsies in UCMD patients (Paco et al., 2013). To investigate if this translated into a systemic increase in protein levels we measured serum levels of the three hormones in sera from patients by ELISA. However, the number of patients in each subgroup for which we had serum samples was small and thus results need to be confirmed.

Determination of serum adipokines showed a significant increase of HMW adiponectin and leptin levels in patients in the intermediate and BM categories. In contrast, HWM adiponectin in DMD patients showed a significant reduction as described in mouse models (Hathout et al., 2014; Abou-Samra et al., 2015).

In obesity, the accumulation of fat leads to a dysregulation of adipokines and decreased circulating levels of adiponectin (Asayama et al., 2003; Kern et al., 2003). In contrast, in this study we observed an increase in HMW adiponectin levels despite the evidence of increased adiposity in the patient cohort. This increase was, however, not significant in the UCMD group which showed the highest degree of body fat. Moreover, circulating HMW adiponectin showed a very strong and consistent positive association with the amount of fat (global and regional) and a negative relationship with the lean mass (global and appendicular).

These observations may indicate that in patients with mutations in collagen VI genes there is another mechanism, independent of the increase in fat mass, which is mediating the increase of circulating adiponectin and perhaps the over-expression of adiponectin gene in muscle that we described (Paco et al., 2013). One may also speculate that patients at the most severe end of the spectrum undergo a reduction in adiponectin similarly to obese patients without collagen VI mutations and that is why their levels were not significantly reduced relative to the control group.

Adiponectin is a hormone strongly related with the regulation of glucose and lipid metabolism in insulin sensitive organs, included skeletal muscle (Caselli, 2014). The decrease in muscle mass in combination with increased adipose tissue described here may be interfering with correct adiponectin-mediated signaling in different steps including adiponectin synthesis, release or receptor sensitivity. According to our results adiponectin circulating levels may represent a valuable indicator of muscle mass loss, however, role of adipokines in the pathophysiology of collagen VI myopathies needs to be further investigated.

In summary, our results show that collagen VI related myopathies are associated with obesity and other alterations in fat tissue mass and distribution and adipokines profile. These should be taken into account in the management of these patients since they represent a risk factor for metabolic and cardiovascular complications. We propose that endocrinological assessment is performed as part of the routine follow up of patients with collagen VI deficiency for early detection of metabolic complications and so that appropriate measures such as dietary control can be applied. Analysis of body composition by DXA is a sensitive and feasible technique to assess the increase in body fat mass and the loss of muscle tissue and as our results show as a quantitative and objective marker of physical performance which could complement other outcome measures to monitor clinical progression and efficacy of therapeutic interventions.

## Author Contributions

MR, LD, MV, JM, AF, MR-K, JD-M, and MO: Study design, acquisition, analysis, and interpretation. CO, CJ, and LG-M: Data acquisition, analysis, and interpretation. All listed authors have contributed to drafting and revising the MS for important intellectual content, have approved the final MS version and agree to be accountable for all aspects of the work.

## Conflict of Interest Statement

The authors declare that the research was conducted in the absence of any commercial or financial relationships that could be construed as a potential conflict of interest.

## Footnotes

1 http://www.who.int/growthref/tools/en/
